# Non-nutritive Sweeteners Induce Hypothalamic ER Stress Causing Abnormal Axon Outgrowth

**DOI:** 10.3389/fendo.2019.00876

**Published:** 2019-12-17

**Authors:** Soyoung Park, Sunjay Sethi, Sebastien G. Bouret

**Affiliations:** ^1^Development Neuroscience Program, Children's Hospital Los Angeles, Los Angeles, CA, United States; ^2^INSERM, Laboratory of Development and Plasticity of the Neuroendocrine Brain, Jean-Pierre Aubert Research Centre, UMR-S 1172, Lille, France; ^3^University of Lille, FHU 1, 000 Days for Health, Lille, France

**Keywords:** aspartame, acesulfame potassium, rebaudioside A, sucralose, hypothalamus, plasticity, development, ER stress

## Abstract

With the prevalence of obesity, non-nutritive sweeteners (NNS) have been widely used as sugar substitutes as they deliver a sweet taste without excessive caloric load. However, it is increasingly recognized that NNS are not inert compounds and may cause long-term metabolic perturbations. Endoplasmic reticulum (ER) stress has emerged as a critical link in the development of obesity and type 2 diabetes. In this study, we investigated the effects of NNS found in common diet beverages (i.e., sucralose, aspartame, acesulfame potassium) and a natural sweetener (i.e., rebaudioside A) on ER stress in the hypothalamic cell line mHypoE-N43/5 *in vivo* and on axonal outgrowth *ex vivo*. Sucralose, aspartame, and acesulfame potassium caused elevated ER stress gene expression in mHypoE-N43/5 cells, with sucralose and acesulfame potassium having the most potent effect. Moreover, acesulfame potassium treatment reduced axon outgrowth from arcuate nucleus explants and this effect was attenuated with the ER stress-relieving drug tauroursodeoxycholic acid. Furthermore, sucralose induced cytotoxicity and acesulfame potassium increases caspase3/7 activity at high concentrations in mHypoE-N43/5 cells. In contrast, rebaudioside A only had moderate effects on hypothalamic ER stress and no adverse effects on axon outgrowth, cytotoxicity, or caspase3/7 activity. Together, our data reveal that commonly consumed NNS cause cellular stress in hypothalamic cells disrupting axon outgrowth and that these biological alterations are not seen with rebaudioside A. These data provide biological plausibility for some NNS to adversely impact metabolic health and identifies rebaudioside A as a sweetener with lower detrimental biological impact on hypothalamic cells.

## Introduction

Dramatic changes in our nutritional environment have most likely contributed to the recent obesity epidemic. This epidemic is not only concerning for an individual's quality of life but also imparts significant health care burdens. Many different methods to battle this issue have gained immense popularity over the past couple of decades, with changes in diet and the use of non-nutritive sweeteners (NNS) being the most popular. Although the concept of consuming less calories by using NNS seems ideal for weight loss and these sweeteners are generally recognized as safe by the US Food and Drug Administration (FDA), there is little evidence regarding their biological actions in metabolically active systems. In fact, recent data indicated that NNS consumption is associated with increased weight gain in rodents (1–3) and humans (4, 5), and glucose intolerance (4–6). Moreover, human data shows the presence of NNS in blood after ingestion and in breast milk (7), suggesting that NNS circulate throughout the body and are likely present during perinatal development. Moreover, NNS, including aspartame, has been shown to cross the blood brain barrier and reach the brain (8). One possible explanation for the lack of clear success in weight loss from NNS consumption may be the possibility that NNS alter central nervous pathways that control energy balance, such as the hypothalamus.

The hypothalamus is involved in the control of food intake and energy expenditure and is a prime target of developmental programming in obesity induced by maternal and perinatal nutritional imbalances (9–11). Multiple studies have shown that perturbations in hypothalamic circuits lead to adverse metabolic outcomes (12, 13), and one potential mechanism for nutritional programming on cellular development is through the activation of endoplasmic reticulum stress (ER) stress (14, 15). ER stress activates a complex intracellular signal transduction pathway called the unfolded protein response (UPR). The UPR is tailored essentially to reestablish ER homeostasis. Previous studies have demonstrated that ER stress and UPR signaling pathway activation play important roles in the development of obesity-induced insulin resistance in type 2 diabetes (16). Obesity caused by high-fat feeding in mice induces ER stress in peripheral tissues and in the hypothalamus, suggesting that metabolic disorders associated with obesity induce ER stress *in vivo* (16–19). Furthermore, relieving ER stress with chemical chaperones, i.e., agents that have the ability to increase ER folding machinery, increases insulin sensitivity and reverses type 2 diabetes in adult leptin-deficient mice and improves leptin sensitivity in adult obese mice fed a high-fat diet (17, 18).

In the present study, we investigated whether commonly used NNS, i.e., sucralose, aspartame, and acesulfame potassium (ACEK), and a natural sweetener, i.e., rebaudioside A, induce hypothalamic ER stress, alter hypothalamic axonal outgrowth, and affect general cell viability. Our study reveals that sucralose, aspartame, and ACEK cause an elevated ER stress response in hypothalamic cells. It also shows that ACEK blunts hypothalamic axonal outgrowth and that this phenomenon appears to involve ER stress pathways. We also report that sucralose and ACEK are cytotoxic while rebaudioside A has no adverse effects on ER stress, axon outgrowth or cytotoxicity.

## Materials and Methods

### Cell Culture Condition

The embryonic mouse hypothalamic cell line mHypoE-N43/5 (20) was cultured in Dulbecco's modified Eagle's medium (Sigma, D5796) supplemented with 10% fetal bovine serum, 100 U/ml penicillin and 100 μg/ml streptomycin at 37°C in 5% CO_2_ and a humidified atmosphere. mHypoE-N43/5 cells were plated out at a density of 6 × 10^5^ cells per well in a 6-wells plate. The following day, medium was changed to culture medium containing either vehicle (saline), or 0.5, 5, 10, or 20 mM of sucralose (Sigma), or aspartame (Sigma), or ACEK (Sigma), or rebaudioside A (Sigma) for 48 h. All non-nutritive sweetener stocks were made in sterile water.

### RNA Extraction and RT-qPCR Analyses

Total RNA was isolated using a PureLink RNA mini kit. cDNA was generated with the High-Capacity cDNA Reverse Transcription Kit (Life Technologies). Quantitative real-time PCR was performed using TaqMan Fast Universal PCR Master Mix and the commercially available TaqMan gene expression primers: *Atf4* (Mm00515324_m1), *Atf6* (Mm01295317_m1), *Xbp1* (Mm00457357_m1), *Bip* (Mm00517691_m1), *Chop* (Mm00492097_m1), and *Gapdh* (Mm99999915_g1). mRNA expression was calculated using the 2^−ΔΔCt^ method after normalization to the expression of the *Gapdh* housekeeping gene. All assays were performed using an Applied Biosystems 7900 HT real-time PCR system.

### Cell Viability Assay

mHypoE-N43/5 cells were cultured in 96-wells black clear bottom cell culture treated plates (Fisher Scientific, Waltham, MA, USA). Cells were exposed to NCSs as described above for 48 h and cell viability was determined by measuring lactate dehydrogenase (LDH) release using the CytoTox 96©Non-Radioactive Cytotoxicity Assay (Promega, Madison, USA) per the manufacturer's directions with a slight modification. The volume of media removed from the wells and thus equivalent volumes used from the kit was reduced to 50 μL. The live cells within the same plate was assessed using the Dojindo Cell Counting Kit-8 (Dojindo Molecular Technologies Inc., Rockville, MD, USA) per the manufacturer's directions. The working reagent was allowed to incubate for 2 h before the plate was read. A subset of mHypoE-N43/5 cells was treated with TUDCA (750 μg/ml) for 48 h to determine effects of TUDCA on cell viability.

### Caspase 3/7 Activity Assay

mHypoE-N43/5 cells were cultured in 96-wells white flat bottom cell culture treated plate (Fisher Scientific). Following 48 h exposure to NNS as described above, caspase 3/7 activity was determined through the Caspase-Glo® 3/7 Assay Systems kit (Promega) following the manufacturer's directions with a slight modification. The volume of media in the well was reduced to 50 μL so 50 μL of the kit reagent was added to the plate. A positive control, staurosporine (50 nM, dissolved in DMSO), was used in these assays with the same exposure time of 48 h. A subset of mHypoE-N43/5 cells was treated with TUDCA (750 μg/ml) for 48 h to determine effects of TUDCA on caspase 3/7 activity.

### Isolated ARH Explant Culture and Image Analysis

Brains were collected from P4 wild-type mice and sectioned at a 200-μm thickness with a vibroslicer as previously described (13, 21). The ARH was then carefully dissected out of each section under a stereomicroscope. Explants were cultured onto a rat tail collagen matrix (BD Bioscience) and each explant was incubated with fresh modified Basal Medium Eagle (Invitrogen) containing either vehicle (saline), or 5 mM of sucralose, or aspartame, or ACEK, or rebaudioside A. A separate set of explants were also pre-treated with TUDCA (750 μg/ml) 4 h prior to add ACEK (5 mM) or vehicle (saline) alone. After 48 h, the explants were fixed in paraformaldehyde and neurites extending from the explants were stained with β-III tubulin (mouse, 1:5,000, Covance) as described previously (21). All animal procedures were conducted in compliance with and approved by the IACUC of the Saban Research Institute of the Children's Hospital of Los Angeles.

Images were then acquired using a Zeiss LSM 710 confocal system. Sections of five different regions of interest (100 × 100 μm) spaced at 100, 200, 300, 400, and 500 μm extending radially from the edge of the ARH explants were acquired with a 10X objective. The image analysis was performed using ImageJ analysis software (NIH), as previously described (13, 21). Briefly, each image plane was binarized to isolate labeled fibers from the background and to compensate for differences in fluorescence intensity. The integrated intensity, which reflects the total number of pixels in the binarized image, was then calculated for each image. This procedure was conducted for each image plane in the stack, and the values for all of the image planes in a stack were summed.

### Statistical Analysis

All values are represented as the mean ± SEM. Statistical analyses were conducted using GraphPad Prism (version 7.0a). Data sets with only two independent groups were analyzed for statistical significance using unpaired two-tailed Student's *t*-test. Data sets with more than two groups were analyzed using one-way analysis of variance (ANOVA) followed by the Bonferroni's Multiple Comparisons test. *P* ≤ 0.05 was considered statistically significant.

## Results

### Non-nutritive Sweeteners Induce ER Stress in Hypothalamic Cells

**Figure 1:**
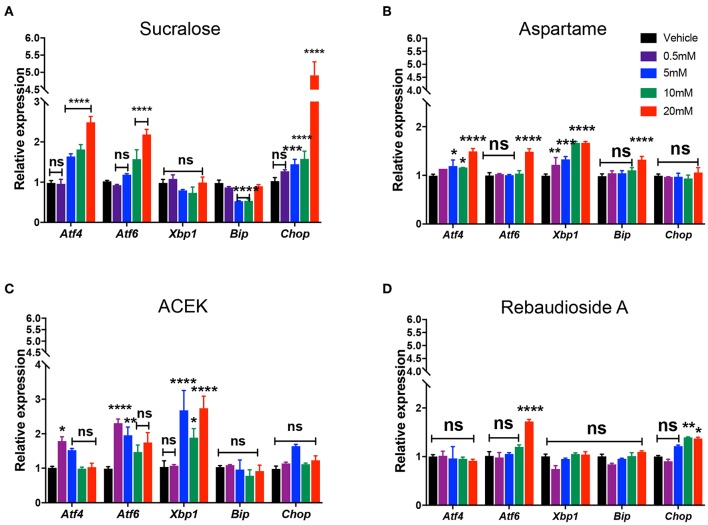
Non-nutritive sweeteners induce ER stress in mouse hypothalamic cell lines. Relative expression of *Atf4, Atf6, Xbp1, Bip*, and *Chop* mRNA in mouse hypothalamic mHypoE-N43/5 cells treated with vehicle (saline) or 0.5, 5, 10, or 20 mM of sucralose **(A)**, aspartame **(B)**, ACEK **(C)**, or rebaudioside A **(D)** for 48 h (*n* = 4 independent cultures per condition). Data are presented as mean ± SEM. *P ≤ 0.05, **P < 0.01, ***P ≤ 0.001, and ****P ≤ 0.0001 vs. all other groups. Statistical significance between groups was determined by two-way ANOVA followed by Bonferroni's Multiple Comparison test.

To examine whether commonly used NNS, sucralose, aspartame, ACEK, and rebaudioside A, directly induced ER stress, we analyzed the expression levels of ER stress markers: activating transcription factor 4 (*Atf4)*, 6 (*Atf6)*, X-box binding protein (*Xbp1)*, glucose regulated protein GRP78 (referred to as *Bip*), and CCAAT-enhancer-binding protein homologous protein (*Chop*) in mouse hypothalamic cell lines, mHypoE-N43/5, at various concentrations (0.5, 5, 10, and 20 mM) of NNS within the range of acceptable daily intake levels (Table 1) (22). *Atf4, Atf6*, and *Chop* were highly induced at concentrations of 5–20 mM sucralose-treated cells but there were no significant changes in *Xbp1* levels (Figure 1A). All of the ER stress genes were slightly upregulated in various concentrations of aspartame-treated cells, except in *Chop* expression (Figure 1B). In addition, ACEK-treated cells showed an increase in most of the ER stress gene expressions except *Bip* and *Chop* (Figure 1C), with 5 mM concentration appearing to be the most ER stress inducible concentration. Notably, rebaudioside A, a metabolite of steviol glycosides synthesis, found in the leaves of Stevia rebaudiana, did not induce significant ER stress gene expression, except in *Atf6* and *Chop* in higher doses (Figure 1D).

**Table 1:** Acceptable daily intake (ADI) of non-nutritive sweeteners from the Joint Commission on Food Additives of the World Health Organization and the Food and Agriculture Organization and representative amount of sweetener in a 12-oz can of diet soda [from (20)].

**Sweetener**	**ADI (mg/kg bw/day)**	**ADI of sweetener based on 70 kg bw (mg)**	**Representative amount of sweetener in 12-oz (354 ml) soda (mg)**	**Sweetener concentration in 12-oz soda (mM)**
Sucralose	15	1,050	68	0.48
Aspartame	40	2,800	187	1.79
ACEK	15	1,050	40 (blended with aspartame)	0.56
Rebaudioside A	4	280	17	0.05

Together, these data indicate that sucralose, aspartame, and ACEK induce hypothalamic ER stress gene expression while rebaudioside A does not induce ER stress when given concentrations of acceptable daily intake. Furthermore, sucralose and ACEK were the most potent NNS at increasing ER stress gene expression.

### ACEK Reduces Axonal Outgrowth and TUDCA Co-treatment Improves Axonal Outgrowth

**Figure 2:**
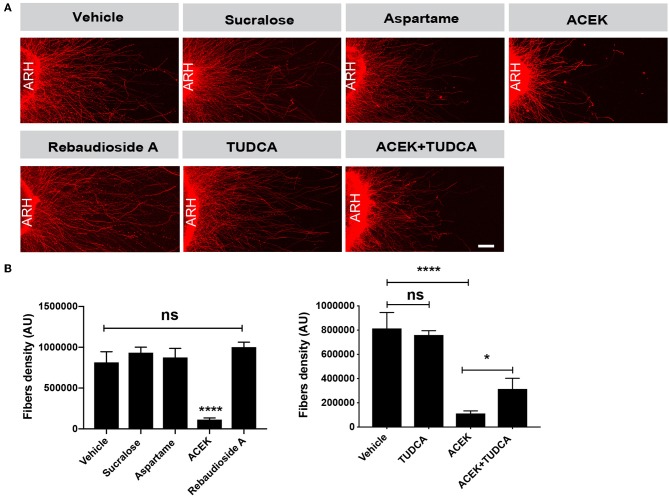
ACEK causes ER stress-induced disruption of arcuate axon outgrowth. Confocal images **(A)** and quantification **(B)** of the density of TUJ1 (neuron-specific class III beta-tubulin)-immunoreactive fibers extending from isolated arcuate nucleus (ARH) explants incubated with incubated with vehicle (saline), or aspartame (5 mM), or sucralose (5 mM), or ACEK (5 mM), or rebaudioside A, TUDCA alone (750 μg/ml), or TUDCA+ACEK (*n* = 6–8 explants per condition). Data are presented as mean ± SEM. *P < 0.05 vs. ACEK treated explants and ****P ≤ 0.0001 vs. control treated explants. Statistical significance was determined by one-way ANOVA followed by Bonferroni's Multiple Comparison test. Scale bars 100 μm.

A variety of signals have been shown to influence hypothalamic axonal outgrowth resulting in metabolic alterations (13, 21, 23, 24). To determine if NNS impact hypothalamic axonal outgrowth, we treated arcuate nucleus (ARH) explants with sucralose, aspartame, ACEK, or rebaudioside A. Briefly, ARH explants were micro-dissected, placed in a collagen matrix, and then exposed to 5 mM of NNS. After 48 h, the density of TUJ1-labeled neurite, neuron-specific class III beta-tubulin, from ARH explants treated with ACEK was approximately 10-fold lower than that of vehicle-treated explants (Figure 2). A more detailed analysis revealed that ACEK not only impaired overall axon density but also reduced the distance that axons grew from the edge of the explant (Supplementary Figure 1). In contrast, sucralose, aspartame and rebaudioside A treatment did not alter arcuate axonal outgrowth (Figure 2). To investigate the importance of ER stress in axon growth, we co-treated explants with tauroursodeoxycholic acid (TUDCA) (750 μg/ml), a chemical chaperone relieving ER stress, with ACEK. TUDCA treatment alone does not alter arcuate axon outgrowth (Figure 2). However, TUDCA treatment improved ACEK-induced changes in arcuate axonal outgrowth (Figure 2).

Together, these data suggest ACEK impairs arcuate axonal outgrowth and density, which can be improved by TUDCA treatment. However, sucralose, aspartame, and rebaudioside A have no adverse effects on arcuate axon outgrowth.

### Sucralose Is Cytotoxic and ACEK Induces Caspase3/7 Activity at High Concentrations

**Figure 3:**
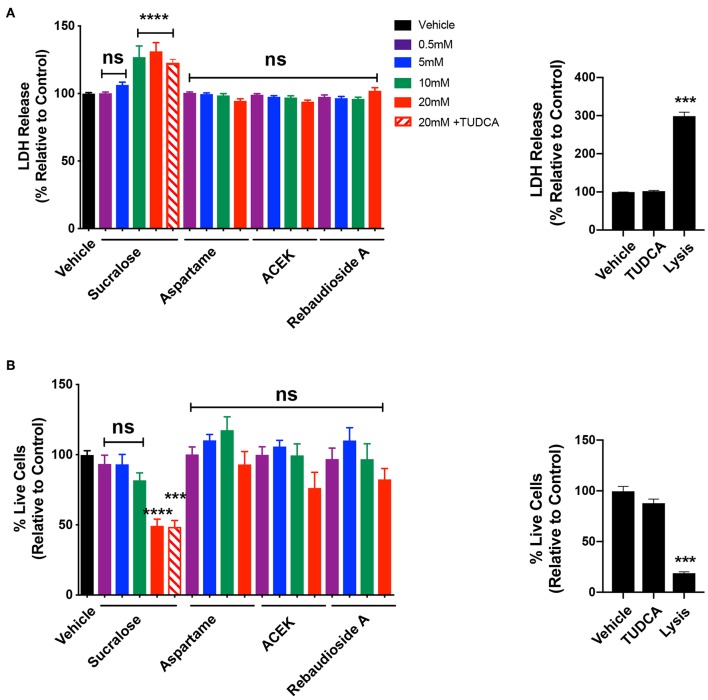
Sucralose decreases cell viability while other non-nutritive sweeteners appear to have no cytotoxic effect. LDH release **(A)** and number of live cells **(B)** in hypothalamic mHypoE-N43/5 cells treated with vehicle (sterile water) or 0.5, 5, 10, or 20 mM of sucralose, aspartame, ACEK, or rebaudioside A, or 20 mM of sucralose with TUDCA (750 μg/ml), or TUDCA alone for 48 h. Lysis solution from the kit was used as a positive control for each assay. Data presented as the mean ± SEM (*n* = 4–6 independent cultures per condition). ***P < 0.001 and ****P ≤ 0.0001 relative to vehicle control as determined using a parametric one-way ANOVA followed by Bonferroni's Multiple Comparison test.

**Figure 4:**
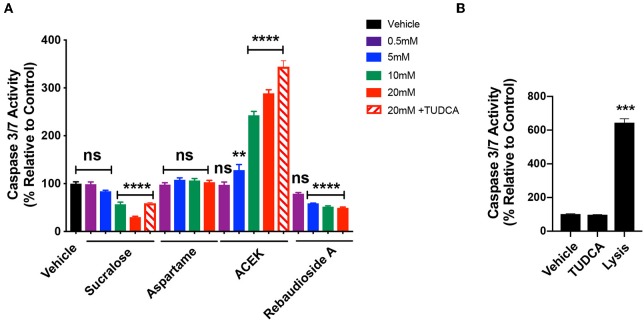
ACEK increases Caspase3/7 activity in hypothalamic cell lines. Caspase 3/7 activity in hypothalamic mHypoE-N43/5 cells treated with vehicle (sterile water) or 0.5, 5, 10, or 20 mM of sucralose, aspartame, ACEK, or rebaudioside A, or 20 mM of sucralose with TUDCA (750 μg/ml), or 20 mM of ACEK with TUDCA (750 μg/ml), or TUDCA alone for 48 h **(A)**. Staurosporine (50 nM) was used as a positive control **(B)**. Data presented as the mean ± SEM (*n* = 4–6 independent cultures per condition). **P < 0.01, ***P < 0.001, and ****P ≤ 0.0001 relative to vehicle control as determined using a parametric one-way ANOVA followed by Bonferroni's Multiple Comparison test.

To test whether common NNS are cytotoxic to hypothalamic cells, we exposed mHypoE-N43/5 cells to a range of concentrations (0.5, 5, 10, and 20 mM) for 48 h and assessed cell viability by measuring the release of the enzyme lactate dehydrogenase (LDH), which is a useful method for detection of necrosis (25) and counted the number of live cells. Sucralose increased LDH release at 10 and 20 mM (Figure 3A) and caused a reduction in the number of live cells at 20 mM (Figure 3B). No other sweetener tested had any effect on LDH release or cell viability outcomes (Figures 3A,B). We also tested whether TUDCA improved the effects of sucralose on cell viability outcomes. However, TUDCA treatment had no effects on sucralose-induced changes in cell viability (Figures 3A,B). As expected, the positive control for these viability assays showed increased LDH release (Figure 3A) and little to no live cells (Figure 3B). Another signaling pathway linked to cellular dysfunction is activation of caspase 3/7 (26). The same experimental paradigm for cell viability was used to assess whether NNS can alter caspase 3/7 activity. ACEK increased caspase activity at 10 and 20 mM (Figure 4A). However, aspartame had no effect on caspase activity while all concentrations of rebaudioside A, as well as 10 and 20 mM of sucralose, led to a decrease in caspase 3/7 activity (Figure 4A). TUDCA treatment at a dose that has previously been shown to improve ER stress (27, 28) had no effect on sucralose- or ACEK-induced changes in caspase 3/7 activity (Figure 4A). The positive control, staurosporine (50 nM), showed significantly enhanced caspase activity (Figure 4B). Notably, TUDCA treatment alone had no effect on cell viability or caspase activity (Figures 3A,B, 4B).

## Discussion

Epidemiological and animal studies have linked NNS consumption with metabolic dysfunction but the underlying mechanisms remain unclear. Recent studies show that NNS affect metabolic traits through gut microbiota (5) or through an imbalance in energy intake and taste perception (29). However, there is a paucity of data regarding the effect of NNS on neuronal systems involved in energy balance. To address this critical data gap, we investigated the direct effect of NNS on hypothalamic ER stress *in vitro*. Our results show that sucralose, aspartame, and ACEK, which are NNS found in common diet beverages, induce the expression of ER stress markers in the hypothalamic mHypoE-N43/5 cells. We further found that ACEK impairs axonal outgrowth from the ARH and that relieving ER stress through TUDCA treatment ameliorates this defect in axonal outgrowth. Moreover, sucralose induced cytotoxicity and ACEK increased caspase 3/7 activity in hypothalamic cell lines. In contrast, the natural sweetener rebaudioside A found in the leaves of Stevia rebaudiana and discovered by Bertoni, only had moderate effects on hypothalamic ER stress, and did not disrupt axon outgrowth or cause cytotoxicity.

Hypothalamic ER stress has recently emerged as a casual factor in the development of central leptin resistance (18, 30, 31). Treatment with chemical chaperones, such as 4-phenyl butyric acid (PBA) and TUDCA, improves protein folding, recovers leptin and insulin sensitivity, normalizes feeding, and reduces body weights (18, 19, 31). Remarkably, we observed an induction in ER stress markers through NNS treatment in the hypothalamic cells. The vast majority of NNS found in common diet beverages induce elevated ER stress in hypothalamic cells, with ACEK being the most potent enhancer of ER stress gene expression causing an increase in *Atf4* and *Atf6* mRNAs with a concentration as little as 0.5 mM. However, the natural NNS rebaudioside A does not induce significant ER stress gene expression, except in *Atf6* and *Chop* at 20 mM.

Cell death, or a loss of cells in a specific brain region, can be damaging even if it is a small percentage such as 10% (32, 33). Based on these observations, it is concerning that sucralose exposure can lead to a significant decrease in cell viability. Surprisingly, sucralose has an opposite effect on caspase 3/7 activity, a pathway that is usually linked to cell death. While these two observations might appear paradoxical, the cell viability data may explain the reason for this apparent discrepancy. There were significantly fewer live cells resulting from sucralose exposure; therefore, sucralose may initiate the apoptotic cascade at a faster rate than the other sweeteners. This would lead to the decrease in live cells and decrease in caspase activity as it has already been activated. Future studies will be needed to examine the kinetics of the effects of NNS on cytotoxicity vs. activation of caspase 3/7. We also found that sucralose treatments from 5–20 mM lead to a marked increase in *Chop* expression, which may lead to apoptosis. It has been demonstrated that the overexpression of *Chop* promotes apoptosis in several cell lines, whereas *Chop*-deficient cells are resistant to ER stress-induced apoptosis (34, 35).

To determine whether NNS also affect hypothalamic axon outgrowth, ARH explants were exposed to a concentration of ACEK that had no effect on cell viability, which could have affected axon outgrowth. The concentration tested in ARH explants was also chosen by selecting one that induced high ER stress gene expression. Arcuate explants exposed to 5 mM of ACEK display attenuated axon outgrowth. Alterations in hypothalamic axon outgrowth has been reported in other models of obesity (13), so it is concerning that similar results were seen with ACEK treatment. Interestingly, rebaudioside A treatment does not change ARH axonal outgrowth compared to control explants. Furthermore, treatment with TUDCA, an alleviator of ER stress, attenuated ACEK-induced perturbation in axon outgrowth, suggesting that ER stress may be one pathway that leads to blunted axonal development. However, the effects of TUDCA appear specific to axon growth, as it had no impact on the decrease in cell viability from sucralose exposure or increase in caspase activity from ACEK exposure.

It remains possible that NNS affect other cellular processes in mHypoE-N43/5 cells. For example, these cells contain machinery responsible for glucose sensing, including glucokinase, glucose transporters, and appropriate ion channels that modulate *Pomc* expression (20). Future studies will be required to examine whether NNS influence glucose sensing in mHypoE-N43/5 cells and alter *Pomc* expression.

A potential concern of this study could be that the NNS concentrations used may or may not reflect physiologically relevant levels in human. However, all of our NNS concentrations tested were within the range of acceptable daily intake levels (Table 1) (22). There is also very minimal human data reporting circulating levels of these NNS and as more of that data is gathered, future studies can be better guided in their concentration selection.

The present study is the first to examine the effect of NNS on hypothalamic ER stress and axon outgrowth. Therefore, although these findings are only *in vitro*, they demonstrate the plausible adverse biological actions NNS may have on hypothalamic neurons. Here, we demonstrate that NNS found in common diet beverages induce hypothalamic ER stress, with sucralose and ACEK in particular inducing ER stress markedly. Additionally, ACEK blunts axon growth. However, rebaudioside A induce significant ER stress gene expression at lower concentrations and does not disrupt axon outgrowth. These results warrant further studies to examine the potential of impact of NNS to on metabolic health and hypothalamic function *in vivo*.

## Data Availability Statement

The datasets generated for this study are available on request to the corresponding author.

## Ethics Statement

The animal study was reviewed and approved by all animal procedures were conducted in compliance with and approved by the IACUC of the Saban Research Institute of the Children's Hospital of Los Angeles.

## Author Contributions

SP conceived, designed, and performed most of experiments and analyzed the data. SS performed cell viability experiments and analyzed the data. SB conceived, designed, and supervised the project. SP, SS, and SB wrote the manuscript. All authors revised and edited the drafted manuscript and approved the final version of manuscript.

### Conflict of Interest

The authors declare that the research was conducted in the absence of any commercial or financial relationships that could be construed as a potential conflict of interest.
