# Supplementary material for: Non-nutritive Sweeteners Induce Hypothalamic ER Stress Causing Abnormal Axon Outgrowth
Source: Front Endocrinol (Lausanne). 2019 Dec 17;10:876. doi: 10.3389/fendo.2019.00876 (PMC6928131; doi:10.3389/fendo.2019.00876)
Supplement: Supplementary file 1 [file Image_1.pdf]

Supplementary Material

Supplementary Figure

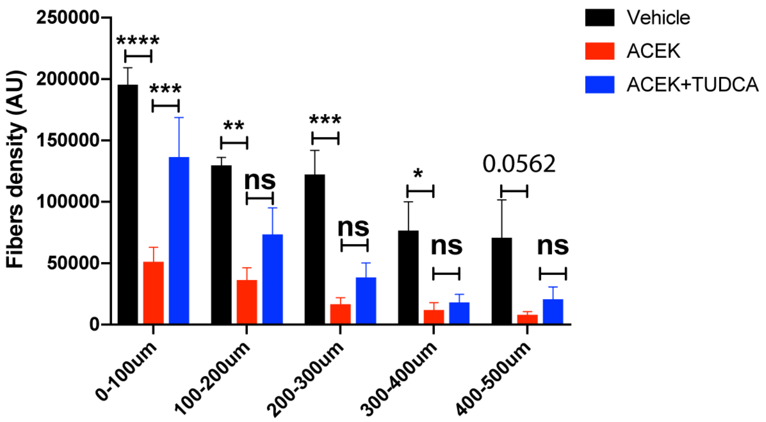

Supplementary Figure 1. Acesulfame potassium reduces axon density and growth from arcuate explants. Quantification of the density of TUJ1 (neuron-specific class III beta-tubulin)-immunoreactive fibers in 5 different regions of interest (100 x 100 µm) spaced at 100, 200, 300, 400, and 500 µm extending radially from the edge of arcuate nucleus explants incubated with vehicle (saline), ACEK (5mM), or ACEK with TUDCA (750 µg/ml) (n = 7-8 explants per condition). Data are presented as mean ± SEM. Statistical significance was determined by two-way ANOVA followed by Bonferroni's Multiple Comparisons test. $*P \leq 0.05$, $**P < 0.01$, $***P \leq 0.001$, and $****P \leq 0.0001$ vs. ACEK treated explants.
